# Application of Venn's diagram in the diagnosis of pleural tuberculosis using IFN-γ, IP-10 and adenosine deaminase

**DOI:** 10.1371/journal.pone.0202481

**Published:** 2018-08-27

**Authors:** Ana Paula Santos, Raquel da Silva Corrêa, Marcelo Ribeiro-Alves, Ana Carolina Oliveira Soares da Silva, Thiago Thomaz Mafort, Janaína Leung, Geraldo Moura Batista Pereira, Luciana Silva Rodrigues, Rogério Rufino

**Affiliations:** 1 Department of Pulmonary Care, Pedro Ernesto University Hospital (HUPE)—State University of Rio de Janeiro (UERJ), Rio de Janeiro, RJ, Brazil; 2 Laboratory of Immunopathology, Medical Sciences Faculty (FCM)—State University of Rio de Janeiro (UERJ), Rio de Janeiro, RJ, Brazil; 3 Laboratory of Clinical Research on STD/AIDS, National Institute of Infectology Evandro Chagas (INI)–Oswaldo Cruz Foundation (FIOCRUZ), Rio de Janeiro, RJ, Brazil; 4 Laboratory of Cellular Microbiology, Oswaldo Cruz Foundation (FIOCRUZ), Rio de Janeiro, RJ, Brazil; University of Cape Town, SOUTH AFRICA

## Abstract

**Background:**

Pleural tuberculosis (PlTB) is the most common extrapulmonary manifestation of this infectious disease which still presents high mortality rates worldwide. Conventional diagnostic tests for PlTB register multiple limitations, including the lack of sensitivity of microbiological methods on pleural specimens and the need of invasive procedures such as pleural biopsy performance. In this scenario, the search for biological markers on pleural fluid (PF) has been the target of several studies as a strategy to overcome the limitations of PlTB diagnosis. This study aims to evaluate the use either isolated or in combination with adenosine deaminase (ADA), interferon-gamma (IFN-γ), interferon-gamma inducible protein of 10-kD (IP-10) levels on PF in order to guide an accurate anti-TB treatment in microbiologically non-confirmed cases.

**Methods and findings:**

Eighty patients presenting pleural effusion under investigation were enrolled in a cross-sectional study conducted at Pedro Ernesto University Hospital, Rio de Janeiro, RJ, Brazil. Peripheral blood (PB) and PF samples collected from all patients were applied to the commercial IFN-γ release assay, QuantiFERON-TB Gold In-Tube, and samples were analyzed for IFN-γ and IP-10 by immunoassays. ADA activity was determined on PF by the colorimetric method. Based on microbiological and histological criteria, patients were categorized as follow: confirmed PlTB (n = 16), non-confirmed PlTB (n = 17) and non-PlTB (n = 47). The *Mycobacterium tuberculosis* antigen-specific production of IFN-γ and IP-10 on PB or PF did not show significant differences. However, the basal levels of these biomarkers, as well as the ADA activity on PF, were significantly increased in confirmed PlTB in comparison to non-PlTB group. Receiver operating characteristics curves were performed and the best cut-off points of these three biomarkers were estimated. Their either isolated or combined performances (sensitivity [Se], specificity [Sp], positive predictive value [PPV], negative predictive value [NPV] and accuracy [Acc]) were determined and applied to Venn's diagrams among the groups. Based on the confirmed PlTB cases, IFN-γ showed the best performance of them at a cut-off point of 2.33 IU/mL (Se = 93.8% and Sp = 97.9%) followed by ADA at a cut-off of 25.80 IU/L (Se = 100% and Sp = 84.8%) and IP-10 (Cut-point = 4,361.90 pg/mL, Se = 75% and Sp = 82.6%). IFN-γ plus ADA _(cut-point: 25.80 IU/L)_ represent the most accurate biomarker combination (98.4%), showing Se = 93.7%, Sp = 100%, PPV = 100% and NPV = 97.9%. When this analysis was applied in non-confirmed PlTB, 15/17 (88.2%) presented at least two positive biomarkers in combination.

**Conclusion:**

IFN-γ, IP-10, and ADA in PlTB effusions are significantly higher than in non-PlTB cases. IFN-γ is an excellent rule-in and rule-out test compared to IP-10 and ADA. The combination of IFN-γ and ADA, in a reviewed cut-off point, showed to be particularly useful to clinicians as their positive results combined prompts immediate treatment for TB while both negative results suggest further investigation.

## Introduction

Tuberculosis (TB) remains a global public health problem ranking above the Acquired Immunodeficiency Syndrome (AIDS) as a leading cause of death between infectious diseases [1]. Among extrapulmonary presentations of the disease, pleural tuberculosis (PlTB) is the most common [2], summing 42% of these cases [3]. Despite its frequency, PlTB remains a challenge for diagnosis due to the paucibacillary nature in patients’ biological specimens and the need for invasive procedures, which are not free from complications, are relatively expensive and time demanding [4–7].

Based on TB’s pathophysiology that represents largely an immunological reaction in which a vast category of cytokines and chemokines are intimately involved, the evaluation of biomarkers on pleural fluid (PF) configures an alternative for TB diagnosis [8–10]. Until now, adenosine deaminase (ADA) is a useful and cost-effective PF marker routinely used in high prevalence settings for diagnosis of PlTB [11]. However, high levels of ADA can also be observed in other types of infections, malignant pleural effusion, and rheumatic diseases, and thus is not specific for PlTB [12].

The role of interferon-gamma release assays (IGRA) adapted to PF for PlTB diagnosis is still under evaluation. Several studies in this line of investigations have been published using the enzyme-linked immunospot (ELISPOT) assay T-SPOT.TB (T-SPOT.TB; Oxford Immunotec Limited, United Kingdom) or the enzyme-linked immunosorbent assay (ELISA) QuantiFERON-TB Gold In-Tube (QFT-GIT; Cellestis Limited, Australia), both IGRA tests approved by World Health Organization to be used only for latent TB diagnosis [13], however showing conflicting results [5, 14–18]. Since that there is an extensive literature [8, 9, 19, 20] regarding the evaluation of PF biomarkers showing that the TB’s lymphocytic pleural effusion presents an increased expression of interferon-γ (IFN-γ) and its inducible chemokines, such as interferon-γ inducible protein of 10-kD (IP-10) [19, 21], we speculate that the application of IGRA could be extremely promising and show considerable adjuvant value on diagnosis of paucibacillary patients.

Given the importance of ADA for PlTB diagnosis and the unsolved determination of the better cut-off value of this marker associated with the advent of new PlTB biomarkers, we aimed to evaluate the use, either isolated or in combination, of ADA, IFN-γ, IP-10 as well as the Mtb-specific immune responses in PF samples based on QFT-GIT system in order to improve the differential diagnosis of PlTB. Receiver operating characteristics (ROC) curves were generated to calculate the best cut-off value for each immunological marker on PF. Further, their performances were estimated in a cohort of patients presenting pleural effusion under investigation and results applied to Venn’s diagrams. The data showed here revealed an effort to apply available diagnostic tools, either alone or in combination, to be used as potential candidates for differential diagnosis of PlTB, especially in cases that do not fit into the TB gold standard methods.

## Material and methods

### Ethics statement

The study protocol was approved by the biomedical research ethics committee of Pedro Ernesto University Hospital, Rio de Janeiro State University (HUPE/UERJ; #1.100.772). All individuals signed a free written informed consent. All samples were fully anonymized before processing to protect the study participants’ identities.

### Study design, samples and data collection

A cross-sectional study was conducted at HUPE/UERJ a tertiary care center at RJ, Brazil. Patients aged ≥ 18 years with pleural effusion under investigation and with an indication of thoracentesis were consecutively enrolled from June 2015 to February 2017. Patients who refused being submitted to the procedure and pregnant women were excluded.

Peripheral blood (PB) was collected and drawn directly into each vacutainer tube provided as part of the QFT-GIT system (QFT-GIT; Cellestis Limited, Australia). Ultrasound-guided thoracentesis was performed by a trained pulmonologist who collected PF which was directly drawn into each tube of QFT-GIT and also sent for routine diagnostic tests, including chemistry panel, total and differential cell count, ADA measurement [22], cytopathology, Xpert MTB/RIF^®^ assay and microbiological analysis [bacteria, fungi and mycobacteria (Lowënstein Jensen solid media)]. Pleural biopsy with Cope’s needle was performed when there were no contraindications and pleural tissue was evaluated for histopathology analysis, acid-fast bacilli (AFB) staining and mycobacterial culture.

We reviewed the medical records of these patients to evaluate physical, clinical and demographic information, medical history and laboratory data. Signs and symptoms included subjective reported presence and duration such as a cough, fever, chest pain, dyspnea, night sweats and weight loss. HIV test was offered to all patients included in the study and their results, as well as the presence of other comorbidities, were also recorded. Radiological finds were based on the chest X-ray and were classified either as: unilateral pleural effusion (UPE) or bilateral pleural effusion (BPE). Data on the respiratory specimen (spontaneous sputum, induced sputum or bronchoalveolar lavage) were collected when available. http://dx.doi.org/10.17504/protocols.io.saceaaw

### Study population and diagnostic criteria

**Confirmed PlTB cases** were defined based on a positive result of the following microbiological and/or histopathological tests on PF or pleural tissue: AFB smear microscopy, mycobacterial culture or Xpert MTB/RIF^®^ and/or evidence of granuloma with or without caseous necrosis.

**Non-confirmed PlTB cases** were consisted of: i) *possible PlTB*–clinical manifestations suggesting TB (fever, chest pain, dyspnea, cough night sweats, hyporexia and/or weight loss), and a lymphocytic and exudative pleural effusion associated with ADA levels above 40 IU/L, followed by a full recovery after at least six months of anti-TB treatment; or ii) e*mpiric PlTB*–cases with clinical manifestations suggesting TB (as previously described above) which do not fill the criteria of confirmed or possible PlTB and that fully recover after at least six months of anti-TB treatment.

**Non-PlTB cases** were defined as those with pleural or pleuropulmonary diseases, excluding active TB based on clinical, laboratory, radiological, microbiological and/or pathological features. Malignant pleural effusions were diagnosed by a positive PF cytologic result or malignant cells identified in the pleural tissue. Even when both of these tests results were negative, malignant effusion was diagnosed when a primary cancer was known to have disseminated and no other cause of pleural effusion was identified. Patients who did not fit the criteria used for PlTB diagnosis defined as above and with unknown cause of pleural effusion were classified as “undefined” (UND) pleural effusion and considered as non-PlTB.

### QuantiFERON-TB Gold In-Tube assay

QFT-GIT was performed in all patients with pleural effusion under investigation. Briefly, one milliliter of both PB and PF were directly drawn in each of the three tubes of QFT-GIT precoated with saline (Nil; negative control) or Mtb-specific antigens [early secretory antigenic target -6 (ESAT-6), culture filtrate protein-10 (CFP-10) and TB 7.7] or mitogen (Mit; positive control) and incubated for 24 h at 37°C. After centrifugation, the supernatant was collected and stored frozen at– 20°C until the IFN-γ determination by an ELISA using QFT-GIT kit according to the manufacturer’s instruction (Cellestis Limited, Australia). IFN-γ-Mtb-specific levels were calculated by subtracting the obtained value with the Nil/control tube. QFT-GIT applied on PB was defined with a positive result when IFN-γ levels in response to Mtb-specific antigens ≥ 0.35 IU/mL and IFN-γ levels in response to mitogen (mitogen minus Nil/control) ≥ 0.5 IU/mL. Indeterminate result was defined as IFN-γ of Nil/control > 8.0 IU/mL or positive control value < 0.5 IU/mL. Results were calculated according to the manufacturer´s software. http://dx.doi.org/10.17504/protocols.io.saseaee

### IP-10 quantification

IP-10 supernatant levels were measured using the Duoset ELISA kit according to the manufacturer’s instructions (R&D Systems Inc, MN, USA). Results were expressed in pg/mL after processing the data with software SoftMax Pro. The lower level of this assay was 31.3 pg/mL while its upper limit was 20,000 pg/mL and readings greater than this were set at 20,000 pg/mL for the purpose of analysis.

### Statistical analysis

In the evaluation of the sociodemographic, clinical and laboratory features among the different groups of individuals, for continuous numerical variables, Kruskal-Wallis ANOVA by Ranks tests was used for assessing the hypothesis that the different samples in the comparison were drawn from the same distribution or from distributions with the same median. Likewise, for categorical nominal variables, Fisher’s exact tests were used in the evaluation of frequencies among the three different groups (confirmed PlTB, non-confirmed PlTB, and non-PlTB) for assessing the hypothesis of independence between the groups of individuals and these variables.

Further, pairwise comparisons of each QFT-GIT parameter mean (Nil/control, Antigen, Mitogen and Antigen minus Nil/control) and ADA dosage were performed by contrasts between confirmed PlTB and non-PlTB groups obtained after both bi- and multivariate linear models fitted by ordinary least square regressions. The decision to use only these two groups (confirmed PlTB and non-PlTB) was based on the concern of possible bias. P-values were corrected by the Tukey Honest Significant Difference (HSD) method. After QFT-GIT parameters and ADA pairwise comparisons, we conducted Holm-Bonferroni Type I error adjustment for multiple comparisons. In order to eliminate sample bias, confounding variables (gender, duration of signs and symptoms, DHL, protein and albumin levels on PF, total, mononuclear (MN) and polymorphonuclear (PMN) cells on PF, chest X-ray, previous TB, history of smoke, and comorbidities as cancer, renal failure and use of immunosuppressive drugs) were selected by bivariate linear models fitted by ordinary least square regressions by backward elimination and were retained in multivariate models if they had any adjusted-P-value < 0.2 in any comparison. The cut-off points of the biomarkers for PlTB diagnosis were calculated from the ROC curve using the Youden method. Again, in order to calculate the best cut-off values, only cases diagnosed using rigid criteria (confirmed TB) were included. Performance of PlTB diagnosis algorithms by the three biomarkers' cut-off points was estimated by its leave-one-out cross-validation (LOOCV) accuracy (Acc), sensitivity (Se), specificity (Sp), positive (PPV) and negative predictive values (NPV), and false-positive and negative ratios with 95% CI. Result classifiers using either each biomarker or their combination were later evaluated, and their results illustrated by Venn’s diagrams. All analysis was performed in software R v. 3.3.1. P-value < 0.05 denoted statistical significance.

## Results

### General characteristics of the study population

**Fig 1:**
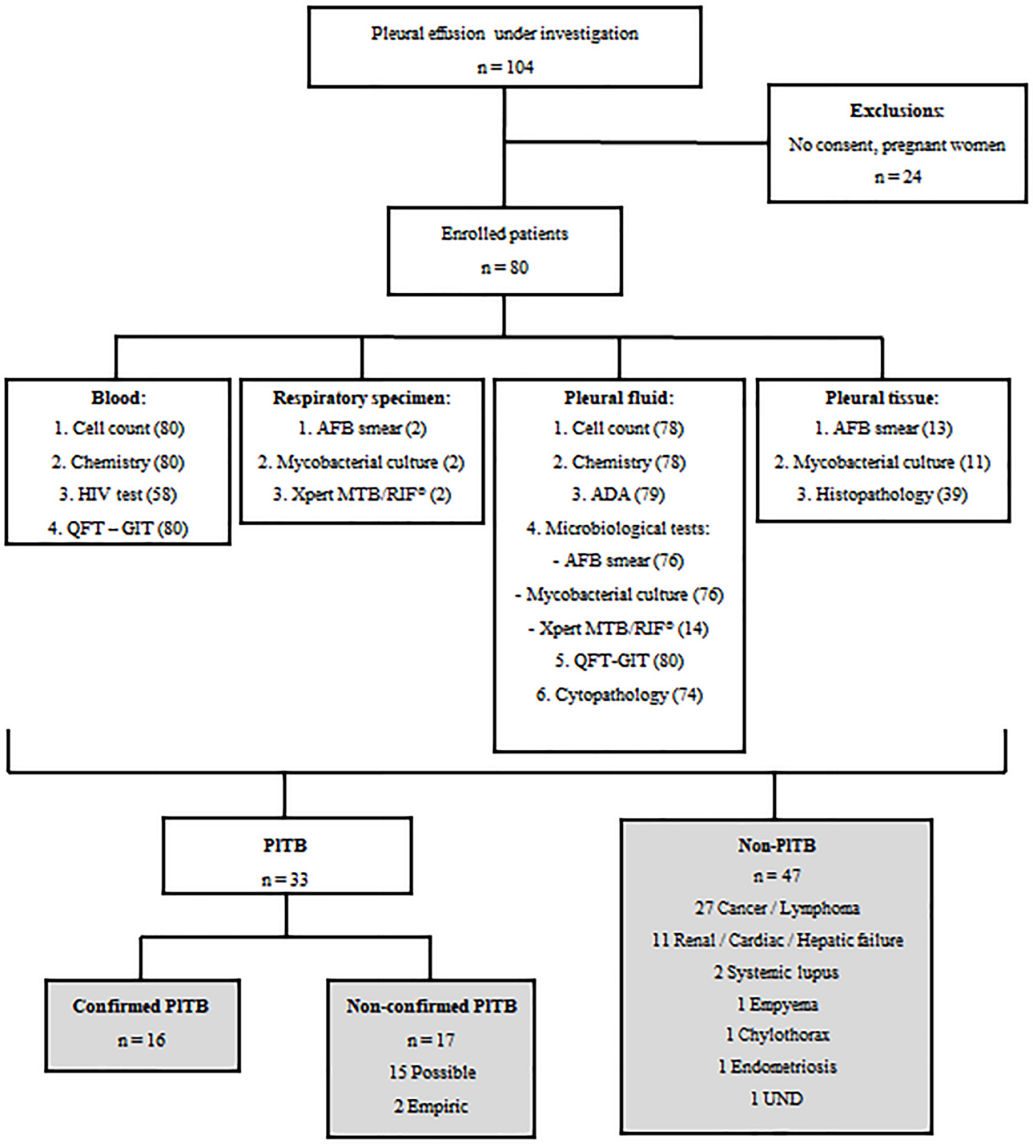
Flow chart of the study design and diagnostic testing performed. QFT-GIT: QuantiFERON-TB Gold in-Tube; AFB: acid-fast bacilli; ADA: adenosine deaminase; TB: Tuberculosis; UND: undefined diagnosis. Numbers in parenthesis refer to the patients submitted to the correspondent diagnostic test. Gray boxes show the final diagnosis and the study groups.

A total of 104 patients were submitted to thoracentesis at HUPE/UERJ between June 2015 and February 2017 in order to determine the pleural effusion etiology. Of these, 80 patients were enrolled in this study by fill the inclusion criteria and a great panel of laboratory investigations were performed, as depicted in Fig 1.

Culture on PF and pleural tissue were performed in 76 and 12 cases, respectively, and histopathological data were available for 39/80 cases (Fig 1). The positivity of this microbiological method in our sample was low, and solid culture on PF and pleural tissue registered values of 9.7% and 22.2%, respectively. Pleural tissue biopsy and the identification of granuloma on histopathological analysis showed a better yield than culture (61.1%). The yield of ADA dosage (> 40 IU/L) was 81.8% and in only 14.3% of PlTB cases, Xpert MTB/RIF^®^ detected Mtb (S1 Table). Thirty-three cases (41%) were diagnosed as PlTB based on the diagnostic criteria previously described [16 confirmed PlTB and 17 non-confirmed PlTB (15 possible and 2 empiric)]. Forty-seven patients (59%) were enrolled as non-PlTB patients: 27 malignancies, 10 renal/cardiac/hepatic failure, 2 empyema, 2 systemic lupus erythematosus, 1 chylothorax, 1 endometriosis and 3 undefined pleural effusion (Fig 1).

Sociodemographic, clinical and diagnostic characteristics of these patients are shown in Table 1. Except for age, a positive history of smoking and arterial hypertension, PlTB and non-PlTB groups were homogeneous. Non-PlTB patients had a higher duration of signs/symptoms until diagnosis when compared to PlTB patients (90 days *vs* 60 days, p = 0.03). Unilateral pleural effusion was the commonest radiological presentation of PlTB group (97%) while non-PlTB cases presented unilateral pleural effusion (68.1%), followed by bilateral pleural effusion (23.4%). Tuberculous PF presented higher levels of lymphocyte percentage (p = 0.01) and total protein (p = 0.04). Confirmed and non-confirmed PlTB did not present statistically different characteristics (Table 1).

**Table 1:** Baseline characteristics of the study population. Socio-demographic, clinical, laboratory and radiological features according to the diagnosis of PlTB (confirmed and non-confirmed cases) and non-PlTB.

Characteristics	Non-PlTB	C-PlTB	NC-PlTB	Non-TB *vs* TB	C-PlTB *vs* NC-PlTB
	(N = 47)	(N = 16)	(N = 17)	*p*-Value	*p*-Value
**Sex, (%)**					
Male	26 (55)	11 (69	12 (71)	0.25	1.0
Female	21 (45)	5 (31)	5 (29)		
**Age, years**					
**Median (IQR)**	62 (49–76)	42.5 (33–49)	45 (26–54)	< 0.0001	0.70
**Smoke, (%)**					
Yes	24 (51)	4 (25)	4 (23.5)	0.03	0.57
**HIV status, (%)**					
Positive	3 (4)	1 (6)	-		0.35
Negative	29 (62)	12 (75)	15 (88)	0.15	
Refuse testing	15 (34)	3 (19)	2 (12)		
**Previous comorbidities, (%)**					
Arterial hypertension	17 (36)	1 (6)	4 (23.5)	0.04	0.33
Diabetes *mellitus*	7 (15)	-	1 (6)	0.13	1.0
Renal failure	3 (6)	-	-	0.26	-
Systemic lupus	1 (2)	-	-	1	-
Viral hepatitis	3 (6)	-	-	0.26	-
Inflammatory bowel disease	-	1 (6)	-	0.41	0.48
Cancer	5 (11)	1 (6)	-	0.39	0.48
Corticosteroids use	-	1 (6)	-	0.24	0.48
Immunosuppressive therapy	3 (6)	-	-	0.26	-
Previous transplant	3 (6)	-	-	0.26	-
Previous tuberculosis	2 (4)	1 (6)	2 (12)	0.66	0.51
**Signs/symptoms, (%)**					
Fever	8 (17)	8 (50)	4 (23.5)	0.14	0.28
Cough	22 (47)	6 (37.5)	8 (47)	0.93	0.66
Chest pain	15 (32)	8 (50)	8 (47)	0.22	0.86
Dyspnea	34 (72)	12 (75)	7 (41)	0.47	0.14
Weight loss	13 (28)	6 (37.5)	6 (35)	0.81	0.86
**Duration of signs/symptoms**					
**Median days (IQR)**	90 (45–195)	60 (27.5–90)	52.5 (30–90)	0.03	0.55
**Pleural fluid, Median (IQR)**					
Total cell count, mm^3^	1,150	2,100	3,600	0.04	0.41
	(500–2600)	(635–3882)	(707–5000)		
Lymphocyte, %	73	90	95	< 0.0001	0.55
	(56–90)	(61–96)	(90–97)		
Total protein, g/dL	4.1	5.55	5.65	< 0.0001	0.35
	(3.6–5.3)	(4.90–6.17)	(4.92–6.15)		
Albumin, g/dL	2.6	2.80	3.00	0.05	0.50
	(1.9–3.0)	(2.60–3.10)	(2.30–3.10)		
DHL, IU/L	185	457	393.5	0.003	0.90
	(137–597)	(214–876)	(259.5–654)		
**ADA, (%)**					
≥ 40 IU/L	2 (4.3)	12 (75)	15 (88.2)	< 0.0001	0.40
< 40 IU/L	44 (93.6)	4 (25)	2 (11.8)		
Missing data	1 (2.1)	-	-		
**Chest X-ray, (%)**					
Unilateral pleural fluid	32 (68.1)	16 (100)	16 (94.1)	0.006	1
Bilateral pleural fluid	11 (23.4)	-	1 (5.9)		
Missing data	4 (8.5)	-	-		
**Peripheral blood QFT-GIT, (%)**					
Positive	11 (23.5)	9 (56.5)	9 (53)	0.01	0.53
Negative	35 (74.5)	6 (37.5)	8 (47)		
Indeterminate	1 (2)	1 (6)	-		

PlTB: Pleural tuberculosis; C-PlTB: Confirmed PlTB; NC-PlTB: Non-confirmed PlTB; IQR: Interquartile range; HIV: Human immunodeficiency virus; DHL: Lactate dehydrogenase; ADA: adenosine deaminase; QFT-GIT: QuantiFERON-TB Gold In-Tube.

ADA activity measurement was performed in 79 out of 80 patients included in the study and was the most frequent diagnostic method used (Fig 1), contributing to PlTB diagnosis in 81.8% of the patients. Using the classical cut-off point of 40 IU/L, ADA positivity showed statistically significant differences between PlTB and non-PlTB positivity frequencies when all cases were considered (S1 Table), while there was no difference between confirmed and non-confirmed PlTB (Table 1).

QFT-GIT assay performed on PB was positive in 18/33 (54.5%) of the PlTB patients, while 11/47 (23.5%) of non-PlTB patients were positive (p = 0.01) (Table 1).

### IFN-γ, IP-10, and ADA measurements in the pleural fluid

Firstly, we decided to determine whether these biomarkers known in the pathophysiology of TB were present at the local site of Mtb infection in our study population. ADA activity was performed directly in PF and the levels of IFN-γ and IP-10 was evaluated after stimulation with Mtb-specific antigens using the QFT-GIT system. In this analysis, we seek to identify possible differences according to the parameters of QFT-GIT performed on PF among confirmed PlTB (n = 16) and non-PlTB (n = 47) groups using an adjusted linear model. The obtained mean values were adjusted to avoid sample bias by selected sets of the following confounding factors: gender, previous TB, cancer, chest X-ray, protein level, PMN percentage and MN percentage for IFN-γ; previous TB, protein level, PMN percentage and MN percentage for IP-10; and protein level, PMN percentage and MN percentage for ADA.

**Fig 2:**
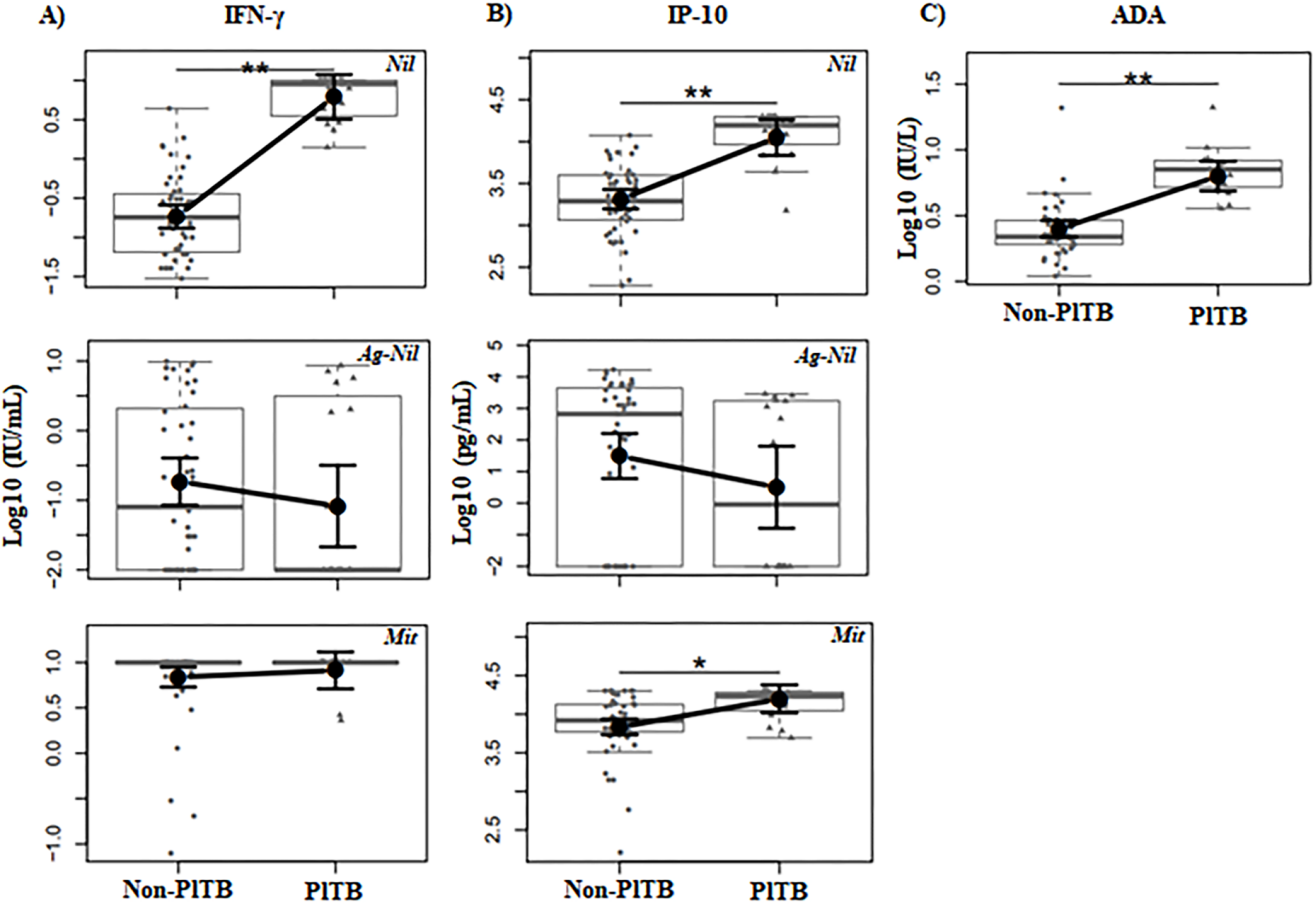
Concentration of IFN-γ, IP-10, and ADA in pleural fluid. (A) IFN-γ and (B) IP-10 were measured in the supernatants of the QFT-GIT system from PF and (C) ADA activity was measured directly in PF. Obtained levels from each biomarker were analyzed in a logarithmic scale and illustrated using boxplots to compare the groups: Non-PlTB (N = 46) and confirmed PlTB (n = 16). Small black dots represent individual cases and box plots represent the interquartile range and sample median (central solid gray line). Bigger black dots and vertical bars represent linear model estimated adjusted means and 95% confidence intervals (CI 95%). Comparisons of means among groups were performed by contrasts/differences obtained after both bi- and multivariate linear models fitted by ordinary least square regressions. The confounders for the parameters illustrated above were: (A) Nil/control (unstimulated) IFN-γ: gender, previous TB, cancer, chest X-ray, protein level, PMN percentage and MN percentage; Ag-Nil IFN-γ: gender, renal failure, and albumin; Mit-stimulated IFN-γ: smoke, chest X-ray, DHL, and albumin; (B) Nil/control (unstimulated) IP-10: previous TB, protein level, PMN percentage, and MN percentage; Ag-Nil IP-10: age, renal failure, and albumin; Mit-stimulated IP-10: previous TB, cancer, smoke, DHL, and albumin; and, (C) for ADA: protein level, PMN percentage, and MN percentage. Nil: Negative/control tube; Ag-Nil: Mtb-specific antigens minus Nil; Mit: Mitogen. * p = 0.001; ** p < 0.0001.

Mtb-specific stimulated PF samples did not demonstrate significant differences regarding both IFN-γ and IP-10 levels between confirmed PlTB and non-PlTB patients in our study population (Fig 2A and 2B). However, the unstimulated or background levels of IFN-γ and IP-10 (Nil/control tube) were higher increased in the PlTB group when compared to non-PTB (p < 0.0001) (Fig 2A and 2B). IFN-γ and IP-10 levels in response to Mtb-specific antigens on PB also did not show significant differences (S1 Fig). ADA activity measurement in PF samples revealed significantly increased levels of this biomarker in confirmed PlTB group (p < 0.0001) in comparison to non-PlTB group, showing five times higher levels on confirmed PlTB (61.05 IU/L in confirmed PlTB *vs* 11.85 IU/L in non-PlTB; p < 0.0001) (Fig 2C).

These data reinforce the idea that IFN-γ, IP-10, and ADA are produced in high levels in Mtb infection site and encourage the use of their baseline levels in the differential diagnosis routine of pleural effusion.

### Performance and diagnostic usefulness of IFN-γ, IP-10, and ADA levels in pleural fluid

Since ADA’s activity is a widely used method in clinical practice for PlTB diagnosis and the vast literature data [8, 9, 19–21] in conjunction with the present study showing the greater levels of IFN-γ and IP-10 in PF, we hypothesize that the combinations of these three biomarkers could improve the TB diagnostic accuracy. First of all, we calculate the best cut-off values for these three biomarkers using confirmed PlTB and non-PlTB (except one case in the non-PlTB group was excluded due to missing data about ADA levels). Moreover, only IFN-γ and IP-10 unstimulated or background (from Nil/control tube) levels were used for the purpose of these analyses as they presented the highest values and statistical differences between the groups.

**Fig 3:**
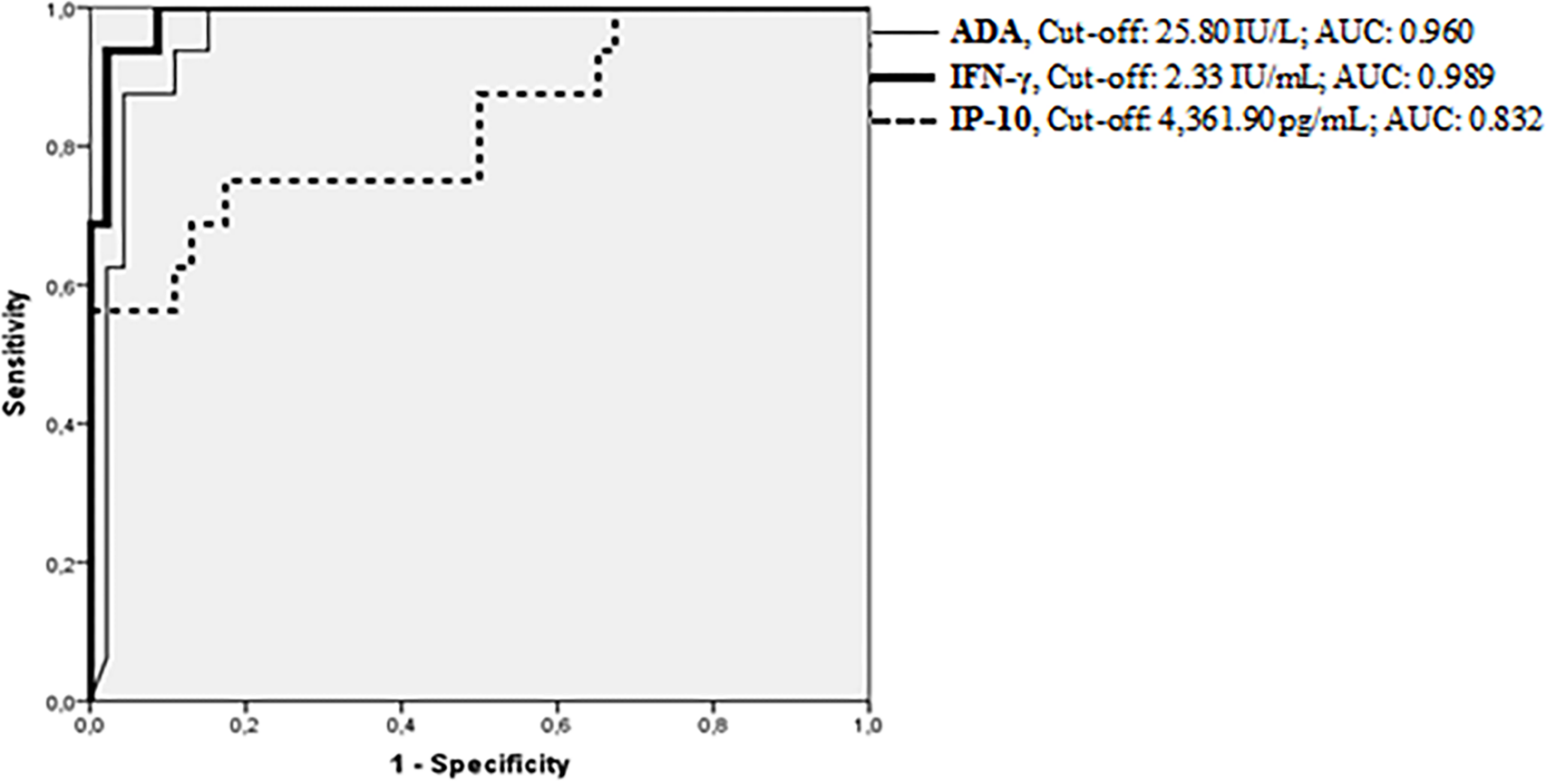
Receiver operating curves of ADA, IFN-γ, and IP-10 in pleural fluid. Receiver operating curves were plotted and each biomarker had its area under the curve (AUC) calculated. The best cut-off value for PlTB diagnosis was established according to Youden’s Index. The statistical analysis was performed using confirmed PlTB (N = 16) and non-PlTB (N = 46).

Receiver operating characteristics curves were performed as shown in Fig 3 and cut-off values were calculated according to the Youden’s Index. IFN-γ registered the highest value of AUC (0.989), followed by ADA (0.960) and IP-10 (0.832). The optimal cut-point for ADA was 25.8 IU/L, which resulted in a Se of 100% (100–100), a Sp of 84.8% (77.2–92.3), a PPV of 69.6% (55.3–83.5), and a NPV of 100% (90.4–100). For IFN-γ, the optimal cut-off value calculated was 2.33 IU/mL, with a Se of 93.7% (84.8–100) and a Sp of 97.8% (94.8–100). Likewise, the optimal IP-10 cut-off value was 4,361.90 pg/mL showing 75% and 82.6% of sensitivity and specificity, respectively (Table 2).

**Table 2:** Performance outcomes of IFN-γ, IP-10, and ADA for the PlTB diagnosis.

	Se	Sp	PPV	NPV	Acc
	(95% CI)	(95% CI)	(95% CI)	(95% CI)	(95% CI)
**Independent IFN-γ**	93.7	97.8	93.7	97.8	96.8
	(84.8–100)	(94.8–100)	(84.8–100)	(94.8–100)	(93.6–99.9)
**Independent IP-10**	75	82.6	60	90.5	80.6
	(58.9–91.1)	(74.6–90.6)	(43.9–76.1)	(84–97)	(73.5–87.7)
**Independent ADA**_**(40 IU/L)**_	75	95.7	85.7	91.7	90.3
	(74.6–75.4)	(95.6–95.8)	(85.4–86)	(91.5–91.8)	(90.2–90.4)
**Independent ADA**_**(25.8 IU/L)**_	100	84.8	69.6	100	88.7
	(100–100)	(77.2–92.3)	(55.6–83.5)	(90.4–100)	(83–94.4)
**IFN-γ (+)**					
**IP-10 (+)**	7	100	100	92	93.5
**ADA**_**(25.8 IU/L)**_ **(+)**	(58.9–91.1)	(100–100)	(100–100)	(86.5–97.4)	(89.1–98)
**IFN-γ (+)**					
**IP-10 (+)**	75	100	100	92	93.5
**ADA**_**(25.8 IU/L)**_ **(-)**	(58.9–91.1)	(100–100)	(100–100)	(86.5–97.4)	(89.1–98)
**IFN-γ (+)**					
**IP-10 (-)**	93.7	100	100	97.9	98.4
**ADA**_**(25.8 IU/L)**_ **(+)**	(84.8–100)	(100–100)	(100–100)	(94.9–100)	(96.1–100)
**IFN-γ (-)**					
**IP-10 (+)**	75	100	100	92	93.5
**ADA**_**(25.8 IU/L)**_ **(+)**	(58.9–91.2)	(100–100)	(100–100)	(86.5–97.4)	(89.1–98)

IFN-γ: interferon-gamma; IP-10: interferon-gamma inducible protein of 10-kD; ADA: adenosine deaminase; Se: Sensitivity; Sp: Specificity; PPV: Positive predictive value; NPV: Negative predictive value; Acc: Accuracy.

Se, Sp, PPV, NPV and Acc are expressed as percentages.

Performance outcomes when confirmed PlTB was compared to non-PlTB patients.

### Combinatorial analysis and Venn’s diagram application in pleural TB diagnosis using ADA _(25.8 IU/L)_, IFN-γ, and IP-10

Although the individual use of these three the immunological markers had shown good performances, analysis in combination of their positivity offered much better results of specificity and PPV, with the capacity of ruling in PlTB as a diagnosis in 100% of our samples when the combination of at least 2 of the biomarkers were positive (Table 2).

None of the confirmed PlTB cases presented isolated positivity of IFN-γ or IP-10. Between them, IP-10 was the least specific (82.6%) showing positive results in 8 non-PlTB cases (5 malignancies, 1 endometriosis, 1 cardiac failure and 1 hepatic failure). Only one case of cardiac failure among the 46 non-PlTB cases was positive for IFN-γ alone (Sp = 97.8%). ADA independent results in their classical cut-point (40IU/L) have shown superior Sp, PPV, NPV, and Acc when compared to the one calculated in the present study (25.8 IU/L; Table 2).

**Fig 4:**
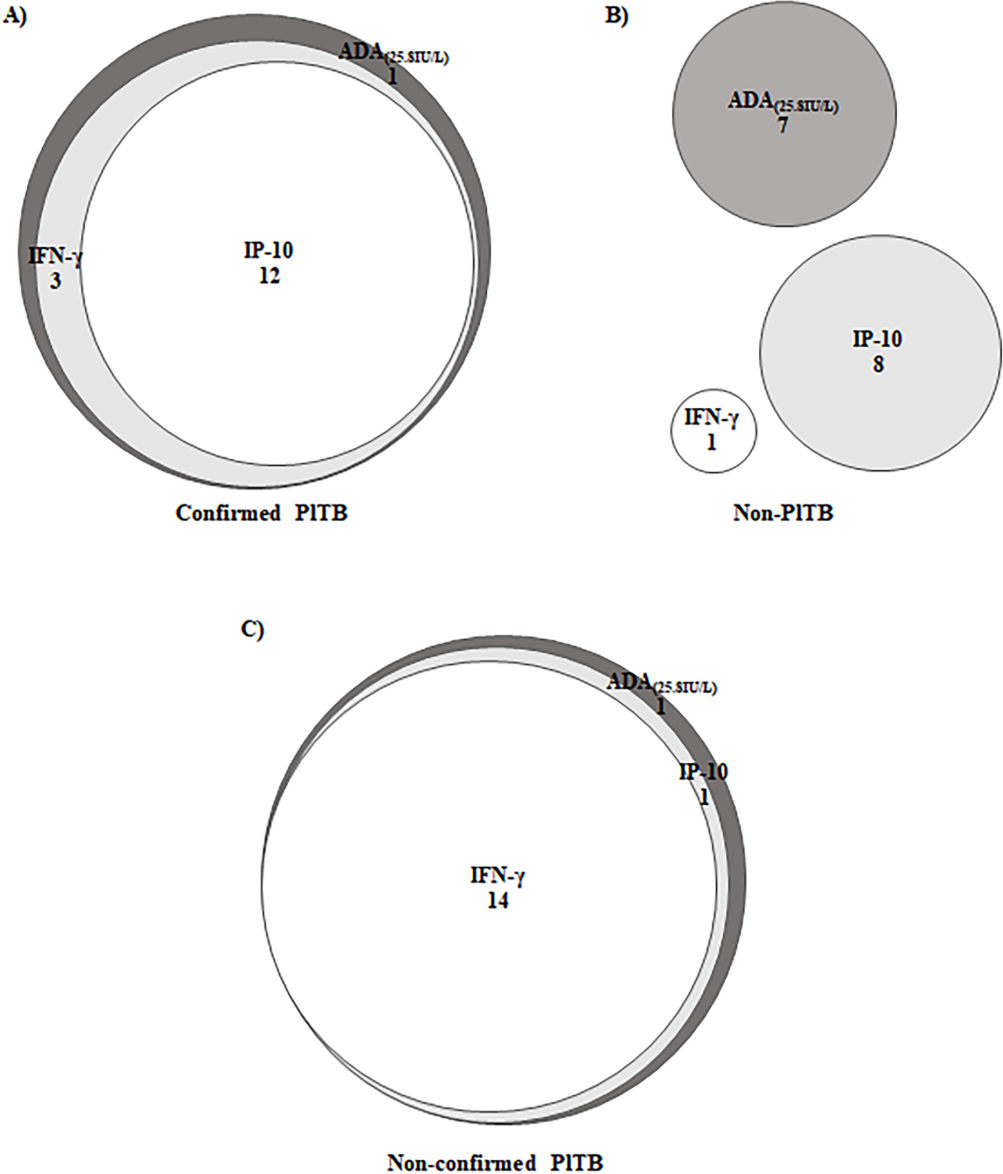
Venn’s diagrams application on PlTB diagnosis using ADA_(25.8 IU/L)_, IFN-γ and IP-10 positivity. A, B and C Venn’s diagrams show the performance of the biomarkers positivity in three different groups of the study: confirmed PlTB (N = 16), non-PlTB (N = 46) and non-confirmed PlTB (N = 17), respectively. IFN-γ: interferon-gamma; IP-10: interferon-gamma inducible protein of 10-kD; ADA: adenosine deaminase; PlTB: Pleural tuberculosis. Numbers indicate the intersection positivity of the biomarkers.

Of the 16 confirmed PlTB cases, 15 (93.7%) were positive using the combination of either two biomarkers among ADA_(25.8IU/L)_, IFN-γ and IP-10 (Fig 4). Noteworthy, none of the non-PlTB was positive (Sp = 100%) using any of these combinations, which resulted in a PPV of 100% and an Acc of 93.5% in our samples, as illustrated in Table 2. Whichever combination of positive biomarkers was done, Sp and PPV achieved 100%, overcoming the lack of specificity of ADA_(25.8IU/mL)_ alone. But the combination of positivity of ADA_(25.8 IU/L)_ plus IFN-γ registered the best performance of all, with an Acc = 98.4% (95% CI 96.1–100%), Se = 93.7%, Sp = 100%, PPV = 100% and NPV = 97.9%. IFN-γ plus IP-10 and ADA_(25.8 IU/L)_ plus IP-10 registered the same behavior, with a lower sensitivity (75%) but a Sp and a PPV of 100% (Table 2).

These results were illustrated using Venn’s diagrams and can be seen in Fig 4. When the 16 confirmed PlTB cases were analyzed, IFN-γ plus IP-10 plus ADA_(25.8 IU/L)_ positivity was registered in 12/16 (87,5%) cases. The intersection of only IFN-γ plus ADA_(25.8 IU/L)_ positivity added more 3 cases, summing 15/16 (93,7%), and in one case ADA_(25.8 IU/L)_ was lonely positive (Fig 4A). In the non-PlTB group, we have observed no intersection among the three biomarkers positivity, which visually represents the specificity of 100% related to possible positivity combinations (Fig 4B). It is worth to note that the isolated positivity of ADA_(25.8 IU/L)_ have resulted in a mistaken diagnosis of 7/46 (15,2%) cases in non-PlTB group (2 systemic erythematous lupus, 2 lymphomas, 1 poorly differentiated cancer, 1 empyema, 1 undefined diagnosis) if only this diagnostic test was used.

When Venn’s diagram was applied in our 17 non-confirmed PlTB cases, IFN-γ plus IP-10 plus ADA_(25.8 IU/L)_ positivity was found in 14/17 (82,3%) cases, IP-10 plus ADA_(25.8 IU/L)_ have included one more case and again, only one case presented the isolated positivity of ADA _(25.8 IU/L)_ (Fig 4C).

## Discussion

To help surpass the limitations of traditional and conventional methods for PlTB diagnoses, such as the lack of sensitivity of microbiological methods on pleural specimens and the need of invasive procedures for pleural biopsy, the search for biological markers on PF has been the target of several studies [23–25]. In our study, we did not observe significant differences regarding IFN-γ and IP-10 production in Mtb-specific PF response between the non-PlTB and confirmed PlTB patients based on the QFT-GIT assay. However, unstimulated or background levels of IFN-γ and IP-10 in the same specimens were significantly higher in PlTB group when compared to non-PlTB. ADA activity, the only biomarker already available in the routine clinical practice for PlTB diagnosis, provided good results when the standardized value of 40 IU/L was used, but the best cut-off value calculated in our sample was 1.6 times less than the one already used. Based on these results, the combinations of positive results of these three biomarkers showed to be useful, especially when IFN-γ and ADA_(25.8 IU/L)_ were both positive, allowing rule-in of PlTB diagnosis in 100% of the cases.

Several studies had already been published about the IGRAs performance on PB or adapted to PF in the PlTB diagnosis [5, 14, 15, 17]. T-SPOT.TB^®^ and QFT-GIT, two IGRAs approved by WHO for latent TB diagnosis [13], have been applied on PF for tuberculous pleurisy diagnosis based on the publication of Wilkinson *et al* [26], which showed that effective T-cells are 15 times higher on PF of PlTB patients than on their PB. So, it was speculated that IGRAs could also be used on active sites of the disease in order to measure the Mtb-specific response according to the amount of cytokines and chemokines produced after the stimulation by the antigens. In our sample, TB group presented a higher percentage of QFT-GIT positivity on PB when compared to non-PlTB (54.5% *vs* 23.5%, p = 0.01). However, the failure of IGRAs on PB in differentiating active from latent TB have shown to be associated with low specificity in regions with high TB burden, as in Brazil. We agree with other previous results that do not suggest the use of IGRAs on PB to differentiate active and latent TB. Our results are in accordance with others which did not find benefits in the use of QFT-GIT on PF, with results of IFN-γ Mtb-specific antigens minus Nil/negative control showing the same behavior in TB and non-PlTB groups. Neither the application of the same method for IP-10 showed significance. These results are possibly explained by the high basal levels of IFN-γ and IP-10 on tuberculous PF, leading to an increase lower than expected after *in vitro* antigen stimulation as they were already and naturally stimulated. Consequently, levels of Mtb-specific antigens minus Nil/negative control biomarkers on PF of TB patients did not show a substantial increase. Moreover, both IFN-γ and IP-10 basal levels proved to be good biomarkers to differentiate TB and non-TB pleural effusions and the use of QFT-GIT kit would only make the diagnosis of PlTB unnecessarily more expensive.

In the last years, PF IFN-γ levels have been reported as a valuable diagnostic tool for PlTB [7, 5, 27–30]. Our results are in accordance with previous reports, including two meta-analysis studies published in 2003 and 2007, which showed a wide range of values for IFN-γ (0.3 to 13 IU/mL) [28, 29]. Other investigations on PlTB diagnosis evaluated the performance IFN-γ levels measured in pg/mL. Ambade *et al* [25] calculated a cut-off of 1,090 pg/mL to differentiate TB pleural effusions from non-PlTB, with a Se = 88%, Sp = 85%. The levels generated by Liu *et al* [30] showed a best performance of this biological marker when compared to ADA to differentiate TB from malignant pleural effusions (IFN-γ: Cut-off = 70 pg/mL, AUC = 0.960, Se = 91.7%, Sp = 97.6%, PPV = 95.7%, NPV = 95.3%; ADA: Cut-off = 30 IU/L, AUC = 0.760, Se = 70.8%, Sp = 95.2%, PPV = 89.5%, NPV = 85.1%). IFN- γ levels applied in a high burden setting for TB [7], presented a better performance than ADA and Xpert MTB/RIF^®^, and based on a cut-off point of 107.7 pg/mL provided a Se of 92.5% and a Sp of 95.9% *versus* a 79% sensitive and 92.7% specific ADA (clinical cut point of 30 IU/L) and a 22.5% sensitive and 98% specific Xpert MTB/RIF^®^, which, in our sample, showed a positivity of 14.3% (S1 Table). Thus, the comparison between different studies is impaired due to differences in the methods of estimations, units and cut-off values applied. Besides that, the results found in the present study are in agreement with previous reports which show IFN-γ as a better marker than ADA for the diagnosis of tuberculous pleurisy, and bringing IP-10 as an alternative method, although its poor performance.

IP-10, a relatively recent studied biomarker is a chemokine involved in trafficking monocytes and activated T-helper type 1 lymphocytes to the TB inflammation site [19, 31]. Previous studies summed 629 patients (304 with pleural TB) in whom IP-10 concentrations measured by ELISA, showed a mean sensitivity and specificity of 84% and 90%, respectively, to discriminate TB and non-PlTB [32]. At a cut-off of 4,361.90 pg/mL, IP-10 was 75% sensitive and 82.6% specific in our sample. Dheda *et al* [33] calculated a ROC-derived cut-point of 28,170 pg/mL for IP-10, which missed approximately 20% of their TB cases and misdiagnosed another 20% of non-PlTB cases. But when a lower cut-point (4,035 pg/mL) was used the NPV achieved 100%, which means that a negative test could exclude TB. Even though the average performance of IP-10, in accordance with Dheda’s results, we presented a considerable number of false positives (5 malignancies, 1 endometriosis, 1 cardiac failure and 1 hepatic failure). In a study published in 2016, TB and malignant pleural effusions were compared using several biomarkers, including IFN-γ and IP-10 [34]. In their sample of 27 patients (5 pleural TB and 22 malignant pleural effusion), IP-10 presented the best AUC when compared to IFN-γ (AUC 0.950 and cut-off 4,005 pg/mL *vs* AUC 0.830 and 100.5 pg/mL), with both presenting the same sensitivity and specificity.

Tuberculous pleurisy is one of the most important differential diagnosis in cases with pleural effusion under investigation with high ADA levels [28]. Our results showed that tuberculous PF presented ADA levels 5 times higher than non-PlTB effusions. A lower ADA cut-off point leads to a sensitivity of 100%, although a fall in specificity to 84.8%. The literature usually reports ADA levels between 30–60 IU/L for PlTB diagnosis [25, 32]. Our value was slightly smaller than this range of values, even though other groups had reported values as low as 10 IU/L, 15 IU/L, 26 IU/L, 30 IU/L and 35 IU/L [2, 28, 35, 36]. There are also other reports suggesting that ADA values can vary according to age, protein, DHL and absolute lymphocyte count on PF, CD4 lymphocyte counts and geographic area [37–41] but these were not our scope. The use of a smaller ADA cut-point lead to seven false positive TB cases (2 systemic erythematous lupus, 2 lymphomas, 1 poorly differentiated cancer, 1 empyema, 1 undefined diagnosis) that could have been diagnosed with PlTB if it was considered alone. These specific conditions are already known as possible false positive when it comes to PlTB diagnosis using ADA and even the use of the standard cut-point of 40 IU/L generated 2 false positives PlTB (1 systemic erythematous lupus and 1 empyema).

The combinatorial analysis of the biomarkers positivity showed an impressing good performance, in particular when IFN-γ plus ADA _(25.8 IU/L)_ was analyzed. In the clinical practice, few studies used two or three assays in combination to improve their isolated diagnostic power for PlTB: QFT-GIT plus polymerase chain reaction [42]; ADA plus lymphocyte percentage on PF [43]; QFT-GIT on PB, ADA and carcinoembryonic antigen (CEA) on PF [2]; T-SPOT.TB on PB and PF combined with ADA on PF [44]. In all cases, the diagnostic yield enhanced when at least two assays were combined. But, despite their performance, our results presented a better behavior, with cut-off point calculated based on a sample with very rigid criteria for PlTB. To the best of our knowledge, we only found one study who tested the combination of IFN-γ plus ADA on PF. Keng *et al* [45], studied 88 patients only with lymphocytic pleural effusions and although they found a specificity of 100% when ADA ≥ 40 IU/L and IFN-γ ≥ 75 pg/mL were combined, their combination was poor accurate and sensitive (41.9% and 79.5%). In the same study, the authors also tested a new cut-off of 15.5 IU/L for ADA, which improved its performance. Maybe, if this lower cut-off was used, like we did in the present study, the combinatorial analysis with IFN-γ could approximate their results with ours. Moreover, we also used neutrophilic pleural effusions, which can be encountered in tuberculous pleurisy, and even so our results were better than theirs.

Finally, the performance of an internal validation of the positive combinations in the non-confirmed PlTB cases showed a sensitivity of 88.2% (15/17), when at least two biomarkers were used. The two exceptions were: i) an empiric case, woman, 22 years old, HIV negative, with an exudative and lymphocytic pleural effusion who had complete clinical and radiological improvement after TB treatment and who presented IFN-γ, IP-10, and ADA_(25.8 IU/L)_ negative results. She also presented 36,000 red blood cells count in PF, which could have influenced the production and consequently, the measurement of these biomarkers; ii) a possible TB, woman, 19 years old, unknown HIV status, with an exudative and lymphocytic pleural effusion, with the highest ADA levels registered in the study (259 IU/L) and false negative results for IFN-γ and IP-10.

Some limitations should be considered in our study. Pleural biopsies were not performed in all patients due to various reasons (old age, coagulopathy, a small amount of PF and refusal of consent the procedure). In two non-PlTB cases (small cell lung cancer and renal failure) AFB smear and culture were lost, both showed all three biomarkers negative. Also, Xpert MTB/RIF^®^ was performed only in 14 cases (seven each group, with only one case of positivity in the TB group), but as already reported, the sensitivity of molecular tests on pleural specimens is low [46–48]. Besides being offered, HIV status was only known in 59 subjects, with only 3 cases of positivity (one undefined pleural effusion with positive ADA and negative IFN-γ and IP-10, one renal failure with all three biomarkers negative, 1 probable TB with all the biomarkers positive). Between the 21 cases of unknown HIV status only one presented an indeterminate QFT-GIT on PB, which means that in 20 cases, besides the unknown immunological condition, they presented a positive response on mitogen tube which represents the positive response of lymphocytes of each patient. Finally, all measurements of IFN-γ and IP-10 were performed using the supernatant from QFT-GIT tubes, and not directly on PF without any processing.

In our study, as in previous data, PlTB patients were more likely to be younger than non-TB ones (Table 1) [7, 15, 30]. Besides the clinical similarities between tuberculous and nontuberculous patients that were possibly related to the magnitude of effusion and to constitutional symptoms [15], time until diagnosis was higher in non-TB patients (Table 1). Tuberculous PF presented higher levels of total cell count, lymphocytic percentage, protein, albumin and DHL levels in the present study (Table 1) and also in previous data [15, 27, 49]. Moreover, we observed that unilateral pleural effusion was the most frequent radiological presentation among cases with TB (Table 1) in accordance to the previous publications [32, 50].

Of the three biomarkers analyzed in this study, IFN-γ showed the best performance. In addition, a lower cut-off point of ADA was identified as a perfect rule-in parameter to PlTB, although showing lower specificity, when combined with IFN-γ it ensured the correct diagnosis in almost 99% of cases. The results should be extrapolated to countries with a similar prevalence of Brazil and be validated in different populations. Although measurements of IFN-γ are relatively expensive and not easily affordable, its use should be considered in future cost-effective studies which could balance the cost of a minimally invasive exam *versus* invasive pleural biopsy procedures, time demanding culture results and the possible adverse effects due to empirical TB treatment.

In conclusion, IFN-γ, IP-10 and ADA levels were significantly higher in the PF from PlTB than in non-PlTB patients. IFN-γ is an excellent rule-in and rule-out test compared to IP-10 and ADA, whether used at its routine value of 40 IU/L or at a lower cut-off point calculated for the purposes of this study. The combination of IFN-γ and ADA _(25.8 IU/L)_ showed to be particularly useful to clinicians as it prompts immediately treatment for TB when both biomarkers are positive or further investigation in cases of both negatives.

## Supporting information

S1 Table Results with classical methods for pleural tuberculosis diagnosis in the study population. (DOCX)

S1 Fig Concentration of IFN-γ and IP-10 in peripheral blood. Both biomarkers were measured on peripheral blood in the supernatants of QFT-GIT. Obtained levels from each biomarker were analyzed in a logarithmic scale and illustrated using boxplots to compare the groups: Non-PlTB (N = 47) and PlTB (n = 33). Small gray dots represent individual cases and box plots represent the interquartile range and sample median (central solid gray line). Bigger black dots and vertical bars represent linear model estimated adjusted means and 95% confidence intervals (CI 95%). PlTB: Pleural tuberculosis; Non-PlTB: Non-pleural tuberculosis; Nil: Negative control tube; Ag: *Mycobacterium tuberculosis*-specific antigen tube; Mit: Mitogen tube; * p = 0.001; ** p < 0.0001.
(TIF)
