# Supplementary material for: Application of Venn's diagram in the diagnosis of pleural tuberculosis using IFN-γ, IP-10 and adenosine deaminase
Source: PLoS One. 2018 Aug 27;13(8):e0202481. doi: 10.1371/journal.pone.0202481 (PMC6110466; doi:10.1371/journal.pone.0202481)
Supplement: S1 Table — (DOCX) [file pone.0202481.s001.docx]

**Supporting Information**

**Table S1 Results with classical methods for pleural tuberculosis diagnosis in the study population**

| **Method** | **Non-Pleural Tuberculosis**  **(%)** | **Pleural Tuberculosis**  **(%)** | ***p*** |
| --- | --- | --- | --- |
| **Mtb culture on PF (N=76)***  Positive  Negative | 0  100 | 9.7  90.3 | 0.1 |
| **Mtb culture on PT (N=11)***  Positive  Negative  Contamination | 0  100  0 | 22.2  66.7  11.1 | 0.04 |
| **Xpert MTB/Rif® on PF (N=14)***  Positive  Negative | 0  100 | 14.3  85.7 | 0.44 |
| **Pleural biopsy (N=39)***  Granuloma  Without granuloma | 4.8  95.2 | 61.1  38.9 | <0.0001 |
| **ADA (N=79)***  Positive  Negative | 4.3  95.7 | 81.8  18.2 | <0.0001 |

Mtb: *Mycobacterium tuberculosis*; PF: Pleural fluid; PT: Pleural tissue; ADA: Adenosine deaminase.

* Number of cases submitted to diagnostic method

|  | **IFN-γ** | **IP-10** |
| --- | --- | --- |
| **Nil** | 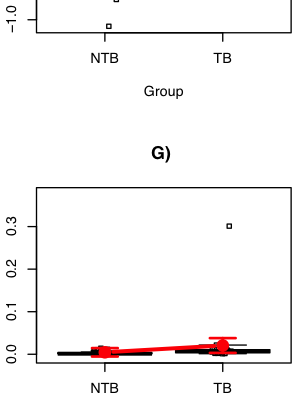 | 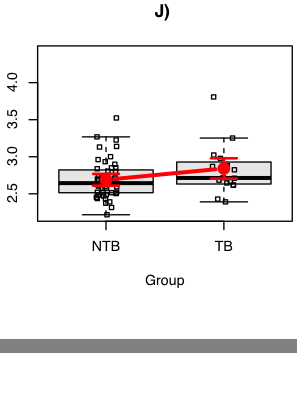 |
| **Ag** | 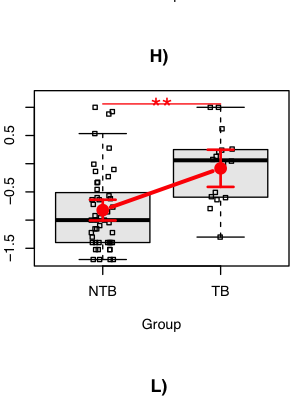 | 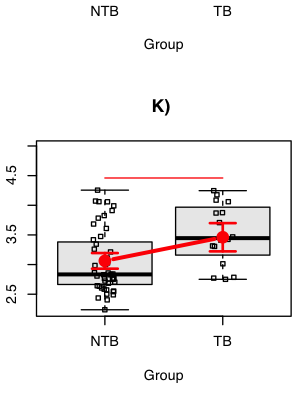 |
| **Mit** | 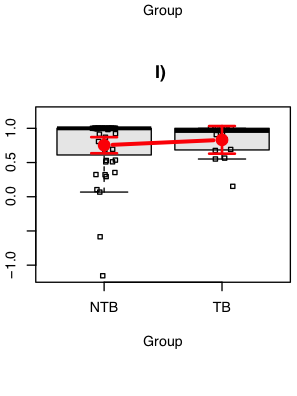 | 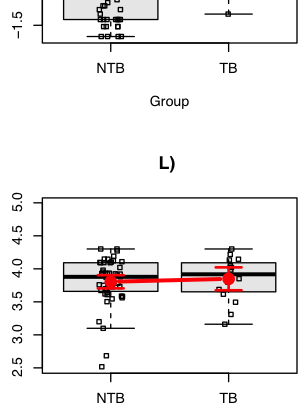 |
| **Ag-Nil** | 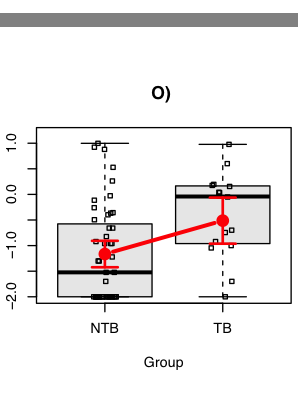 | 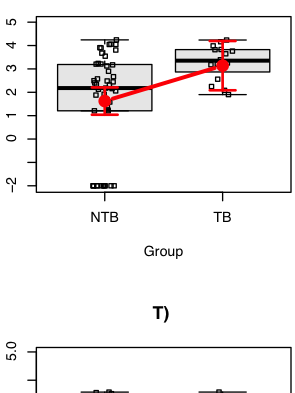 |

**Figure S1 Concentration of IFN-γ, IP-10, and ADA in peripheral blood.** Both biomarkers were measured on peripheral blood in the supernatants of QFT-GIT. Obtained levels from each biomarker were analyzed in a logarithmic scale and illustrated using boxplots to compare the groups: Non-PlTB (N = 47) and PlTB (n=33). Small black dots represent individual cases and box plots represent the interquartile range and sample median (central solid gray line). Bigger red dots and vertical bars represent linear model estimated adjusted means and 95% confidence intervals (CI 95%). PlTB: Pleural tuberculosis; Non-PlTB: Non-pleural tuberculosis; Nil: Negative control tube; Ag: *Mycobacterium tuberculosis*-specific antigen tube; Mit: Mitogen tube; * p = 0.001; ** p < 0.0001.
