# Supplementary figures and images for: Application of Venn's diagram in the diagnosis of pleural tuberculosis using IFN-γ, IP-10 and adenosine deaminase
Source: PLoS One. 2018 Aug 27;13(8):e0202481. doi: 10.1371/journal.pone.0202481 (PMC6110466; doi:10.1371/journal.pone.0202481)

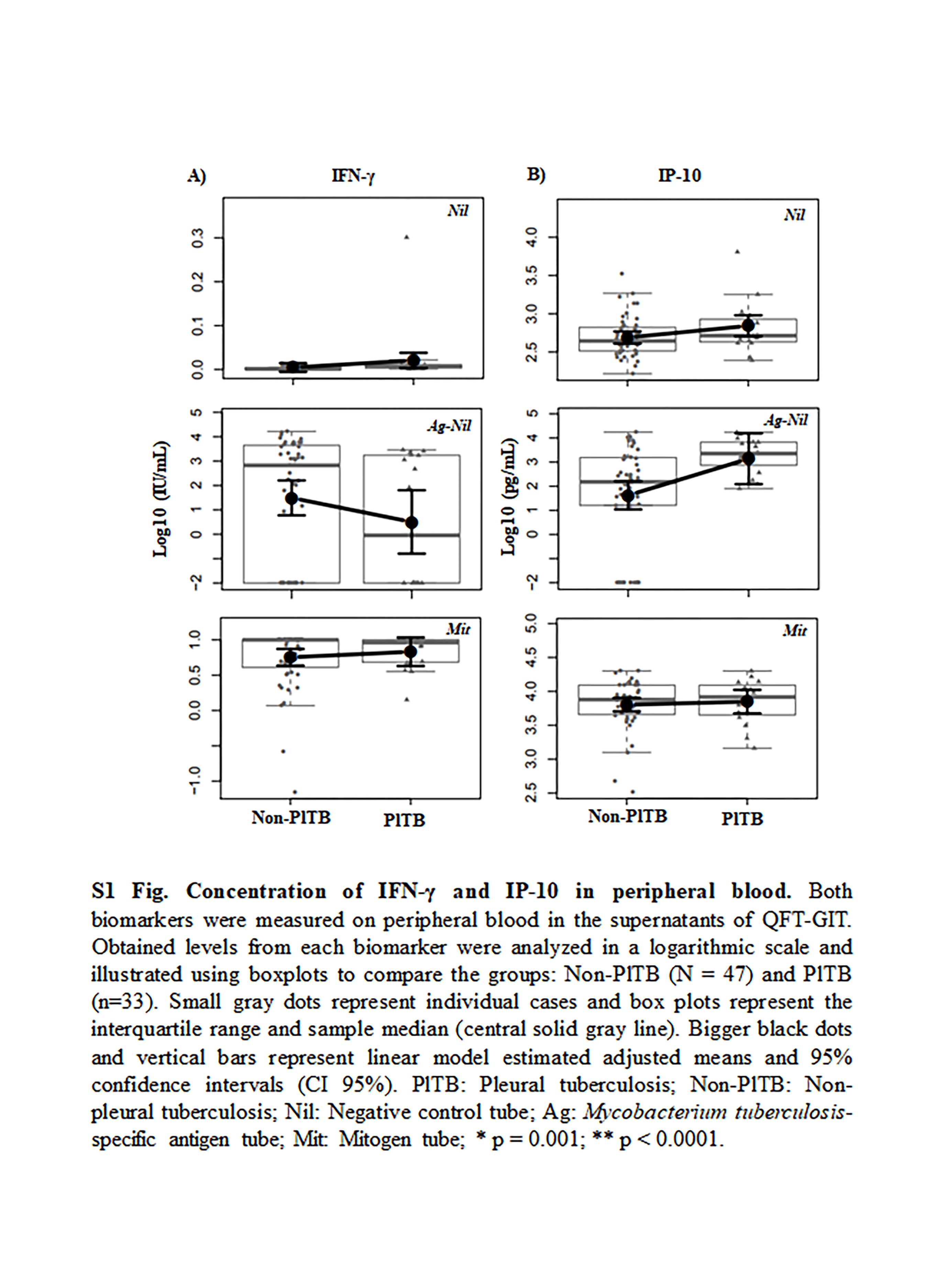

Supplement: S1 Fig — Both biomarkers were measured on peripheral blood in the supernatants of QFT-GIT. Obtained levels from each biomarker were analyzed in a logarithmic scale and illustrated using boxplots to compare the groups: Non-PlTB (N = 47) and PlTB (n = 33). Small gray dots represent individual cases and box plots represent the interquartile range and sample median (central solid gray line). Bigger black dots and vertical bars represent linear model estimated adjusted means and 95% confidence intervals (CI 95%). PlTB: Pleural tuberculosis; Non-PlTB: Non-pleural tuberculosis; Nil: Negative control tube; Ag: Mycobacterium tuberculosis-specific antigen tube; Mit: Mitogen tube; * p = 0.001; ** p < 0.0001. (TIF) [file pone.0202481.s002.tif]
